# Neural activity patterns between different executive tasks are more similar in adulthood than in adolescence

**DOI:** 10.1002/brb3.1063

**Published:** 2018-07-26

**Authors:** Mona Moisala, Viljami Salmela, Synnove Carlson, Katariina Salmela‐Aro, Kirsti Lonka, Kai Hakkarainen, Kimmo Alho

**Affiliations:** ^1^ Department of Psychology and Logopedics Faculty of Medicine University of Helsinki Helsinki Finland; ^2^ AMI Centre Aalto NeuroImaging Aalto University School of Science Espoo Finland; ^3^ Department of Neuroscience and Biomedical Engineering Aalto University School of Science Espoo Finland; ^4^ Department of Physiology Faculty of Medicine University of Helsinki Helsinki Finland; ^5^ Faculty of Educational Sciences University of Helsinki Helsinki Finland; ^6^ Institute of Education University College London London UK; ^7^ Optentia Research Focus Area North‐West University Vanderbijlpark South Africa

**Keywords:** adolescence, brain imaging, development, executive functions, fMRI, functional connectivity

## Abstract

**Background:**

Adolescence is a time of ongoing neural maturation and cognitive development, especially regarding executive functions. In the current study, age‐related differences in the neural correlates of different executive functions were tracked by comparing three age groups consisting of adolescents and young adults.

**Methods:**

Brain activity was measured with functional magnetic resonance imaging (fMRI) from 167 human participants (13‐ to 14‐year‐old middle adolescents, 16‐ to 17‐year‐old late adolescents and 20‐ to 24‐year‐old young adults; 80 female, 87 male) while they performed attention and working memory tasks. The tasks were designed to tap into four putative sub‐processes of executive function: division of attention, inhibition of distractors, working memory, and attention switching.

**Results:**

Behaviorally, our results demonstrated superior task performance in older participants across all task types. When brain activity was examined, young adult participants demonstrated a greater degree of overlap between brain regions recruited by the different executive tasks than adolescent participants. Similarly, functional connectivity between frontoparietal cortical regions was less task specific in the young adult participants than in adolescent participants.

**Conclusions:**

Together, these results demonstrate that the similarity between different executive processes in terms of both neural recruitment and functional connectivity increases with age from middle adolescence to early adulthood, possibly contributing to age‐related behavioral improvements in executive functioning. These developmental changes in brain recruitment may reflect a more homogenous morphological organization between process‐specific neural networks, increased reliance on a more domain‐general network involved in executive processing, or developmental changes in cognitive strategy.

## 1 INTRODUCTION

Both functional and structural aspects of the brain continue to mature throughout adolescence (Paus, 2005), resulting in development reflected in behavior, cognition, and brain architecture. A key target of development during the adolescent period is suggested to be executive functioning, a set of cognitive processes needed in the coordination and control of goal‐directed behavior (Luria, 1966). Although evidence suggests that executive functioning can be thought of as a unitary, subordinate system of cognitive control (Niendam et al., 2012), low correlations in task performance between different executive task types imply that executive functions form a collection of at least partly distinguishable top‐down mental processes (Duncan, Johnson, Swales, & Freer, 1997; Shallice & Burgess, 1996). Although varying conceptualizations have been offered regarding the exact nature of executive sub‐processes, most accounts describe three core functions: inhibition (including behavioral inhibition, selective attention and cognitive inhibition), monitoring and updating of working memory representations, and cognitive flexibility or set shifting (e.g., Diamond, 2013; Lehto, Juujärvi, Kooistra, & Pulkkinen, 2003; Miyake et al., 2000).

Executive functioning improves throughout adolescence, so that different rates of improvement are observed for different executive processes (Best, Miller, & Naglieri, 2011; De Luca et al., 2003; Diamond, 2013; Gur et al., 2012; Luciana, Conklin, Cooper, & Yarger, 2005; Luna, Garver, Urban, Lazar, & Sweeney, 2004; Taylor, Barker, Heavey, & McHale, 2015). Age‐related behavioral enhancements in executive functioning are thought to result from gradual changes in white matter density and organization due to myelination, as well as from changes in gray matter volume due to synaptic pruning in the brain (Blakemore & Choudhury, 2006; Gogtay et al., 2004). In terms of brain activity, previous neurodevelopmental studies most often report enhanced neural recruitment with age during executive task performance (Casey, Giedd, & Thomas, 2000; Rubia, 2013). For example, studies have shown age‐related behavioral improvements to be coupled with increases in the magnitude of neural activity during tasks requiring working memory (Geier, Garver, Terwilliger, & Luna, 2009; Jolles, Kleibeuker, Rombouts, & Crone, 2011; Satterthwaite et al., 2013) and inhibition (Durston et al., 2006; Marsh et al., 2006; Rubia et al., 2013; Vink et al., 2014). Conflicting results have also been obtained, however, with some studies reporting more task‐related activity in child and adolescent participants than in adults (Booth et al., 2003; Ordaz, Foran, Velanova, & Luna, 2013; Somerville, Hare, & Casey, 2011). Further, it has also been suggested that age‐related changes are evident more in the temporal dynamics of brain region recruitment, rather than in the magnitude or location of that recruitment (Wendelken, Munakata, Baym, Souza, & Bunge, 2012). Given these diverse results, defining what constitutes “mature or adult brain activity” during cognitive processing is difficult and cannot simply be defined in terms of the amount of activity in a specific region (Crone & Steinbeis, 2017; Somerville, 2016). An adolescent may, for example, utilize a different cognitive strategy than an adult to accomplish a task, and thus also recruit different neural networks. Furthermore, it may be that the cognitive architecture of executive functions changes with age, thus resulting in differences in neural recruitment patterns. For example, it has been suggested that executive functions may shift from a unitary control process toward a more differentiated function during childhood development (Brydges, Fox, Reid, & Anderson, 2014).

Measures of functional connectivity have also been used to study brain maturation, as they provide insight into the functional integration of brain circuits and causal influences between network nodes, rather than just activity patterns of isolated regions. Developmental studies most often report increasing functional connectivity with age during tasks requiring cognitive control (e.g., Stevens, Kiehl, Pearlson, & Calhoun, 2007; Supekar & Menon, 2012; Washington & VanMeter, 2015). Adolescent brain development has also been shown to result in shifts from local to more distal connectivity (Keulers, Goulas, Jolles, & Stiers, 2012). Together these results seem to suggest that cognitive development is accompanied by a reconfiguration of the hierarchical, modular organization of brain networks (Stevens, 2016).

In this study, our aim was to examine differences in the neural correlates of executive functions between adolescence and young adulthood, in terms of regional activity patterns and functional connectivity. To this end, 167 adolescent and young adult participants (13–24‐year‐olds) performed attention and working memory tasks while brain activity was measured with fMRI. The experimental tasks tapped into four key executive functions: the ability to (a) divide attention between two sensory stimuli simultaneously, (b) ignore or inhibit the processing of distractors, (c) retain and manipulate information in working memory, and (d) switch attention between different sensory modalities. Behaviorally, we expected to see superior performance in older participants, as improvements in cognitive performance are known to occur during adolescence (Levin et al., 1991; Paus, 2005). In terms of brain activity, the different executive tasks were expected to recruit regions in parietal cortical regions and in lateral and medial prefrontal regions, as demonstrated by our earlier work (Moisala et al., 2015, 2017). Functional connectivity between these frontoparietal nodes was expected to be similar irrespective of the specific demands of each task type, as the components of this “frontoparietal control system” (Vincent, Kahn, Snyder, Raichle, & Buckner, 2008) are co‐active in a wide variety of task domains (Duncan, 2010; Fedorenko, Duncan, & Kanwisher, 2013) and are known to subserve a variety of executive functions (Niendam et al., 2012). Moreover, age‐related differences in cortical activity and functional connectivity overlap between different executive task types were of special interest.

The current study provides unique insight into neural maturation, as studies directly comparing the spatial overlap of neural activity patterns elicited by different executive tasks between different age groups have not been previously conducted. Similarly, although age‐related changes in brain functional connectivity during executive task performance have been previously explored, these studies have not directly compared the degree of similarity of frontoparietal functional connectivity between different executive task types. The results of the current study therefore offer the possibility to reveal completely novel aspects of neural and cognitive maturation during adolescence.

## 2 METHODS

### 2.1 Participants

The participants consisted of three different age cohorts: 13‐ to 14‐year‐old (middle adolescents; *n* = 736) and 16‐ to 17‐year‐old (late adolescents; *n* = 1,130) pupils and 20‐ to 24‐year‐old (young adults; *n* = 1,111) university students. The participants were selected from a sample of 2,977 respondents, who had filled out a questionnaire relating to the use of digital technologies as a part of the research project titled Mind the Gap between Digital Natives and Educational Practices (2013–2016). The questionnaire included a Sociodigital Participation (SDP) inventory assessing various dimensions of technology‐mediated practices in everyday life. Using a latent profile analysis (Vermunt & Magidson, 2002), all questionnaire respondents (each cohort separately) were first grouped into three profiles representing their SDP practices for the purpose of studying the effects of technology use on cognitive functioning (Moisala et al., 2016, 2017): basic participants (control), gaming‐oriented participators, and creative participators. These SDP profiles were not utilized in the current study, as our aim was only to examine developmental effects on cognitive performance and brain activity. Respondents ineligible for an fMRI measurement were screened out (i.e., respondents with metal implants, braces, tattoos), as well as respondents with any learning difficulties. Respondents with notably poor school performance with a self‐reported grade point average (GPA) below 7 on a 4‐ to‐10‐point scale system were excluded. GPA was based on the most recent diploma for the middle adolescent cohort, the junior high school diploma for the late adolescent cohort, and the high school diploma for the young adult cohort. In the Finnish education system, the GPAs obtained from these different education levels are directly comparable. Respondents who demonstrated the highest likelihood of belonging to their respective SDP profiles were then asked to participate in the fMRI study. As a result, brain activity and performance of 173 participants were measured for the study, of which 167 had good data quality and no technical difficulties during fMRI measurement (Table 1). The same dataset was used in previously published studies linking technologically mediated activities to brain functioning, that is, media multitasking to increased distractibility and right prefrontal cortical activity (Moisala et al., 2016), and gaming to enhanced working memory performance and frontoparietal cortical recruitment (Moisala et al., 2017). All participants were native Finnish speakers with normal hearing, normal or corrected‐to‐normal vision, and no self‐reported history of psychiatric or neurological illnesses. An informed written consent was obtained from each participant (and from a guardian in the case of underage participants) before the experiment. The experimental protocol was approved by the Ethics Committee for Gynaecology and Obstetrics, Pediatrics and Psychiatry of The Hospital District of Helsinki and Uusimaa, and it was conducted in accordance with the principles of the Declaration of Helsinki.

**Table 1:** Participant characteristics

	13–14 years (*n* = 57)		16–17 years (*n* = 57)		20–24 years (*n* = 53)	
	Age (±*SD*)	GPA (±*SD*)	Age (±*SD*)	GPA (±*SD*)	Age (±*SD*)	GPA (±*SD*)
Female (*n* = 80)	13.1 (±0.4)	8.6 (±0.5)	16.6 (±0.5)	9.1 (±0.5)	20.6 (±1.3)	8.9 (±0.7)
Male (*n* = 87)	13.3 (±0.5)	8.4 (±0.7)	16.6 (±0.5)	8.8 (±0.7)	21.9 (±0.9)	8.3 (±0.9)

*Note*

Means and standard deviations (*SD*s) of ages and self‐reported grade point averages (GPAs) for all three age cohorts and separately for females and males.

### 2.2 Stimuli

The stimuli in the attention task comprised of visual and auditory sentences that were either semantically congruent or incongruent. The incongruent sentences were created by taking a subset of the congruent sentences (e.g., “*This morning I ate a bowl of cereal*”) and replacing the last word of the sentences with a semantically incongruent word (e.g., “*This morning I ate a bowl of shoes*”). In addition, excerpts of instrumental music were used as distractors in attention tasks. In the *n*‐back task, the visual stimuli were written vowels a, e, u, and y, and the auditory stimuli were the same vowels as spoken in Finnish. For more details on the stimuli, please see our previous studies (Moisala et al., 2016, 2017).

The visual stimuli in both task types were presented on a video screen projected onto a mirror mounted on the head coil. All auditory stimuli were high‐pass filtered with a cutoff at 100 Hz and low‐pass filtered with a cutoff at 7,000 Hz. Auditory stimuli were delivered through insert earphones (Sensimetrics, Malden, MA, USA). Their intensity was individually set to a loud, but pleasant level, while noise from the MRI scanner was attenuated by earplugs integrated into earphones, circumaural ear protectors (Bilsom Mach 1, Bacou‐Dalloz Inc., Smithfield, Rhode Island, USA), and viscoelastic mattresses around the head coil. All adjustments to the auditory stimuli were made using Audacity (http://audacity.sourceforge.net) and MATLAB (Mathworks Inc., Natick, MA, USA) softwares.

### 2.3 fMRI/MRI data acquisition

Functional brain imaging was carried out with a 3 T MAGNETOM Skyra whole‐body scanner (Siemens Healthcare, Erlangen, Germany) using a 20‐channel head coil. The functional echo planar (EPI) images were acquired with an imaging area consisting of 43 contiguous oblique axial slices (TR 2,500 ms, TE 32 ms, flip angle 75°, voxel matrix 64 × 64, field of view 20 cm, slice thickness 3.0 mm, in‐plane resolution 3.1 mm × 3.1 mm × 3.0 mm). Image acquisition was performed at a constant rate (i.e., image acquisition was not jittered), but was asynchronized with stimulus onsets. For the attention task, three functional runs of 222 volumes (including 4 initial dummy volumes) were measured for each participant. The duration of one run was 11 min. For the *n*‐back task, two functional runs of 155 volumes (including 4 initial dummy volumes) were measured. The duration of one run was 7 min.

High‐resolution anatomical images (MPRAGE, voxel matrix 256 × 256, in‐plane resolution 1 mm × 1 mm × 1 mm) were acquired from each participant midway through the measurement session.

### 2.4 Experimental design

**Figure 1:**
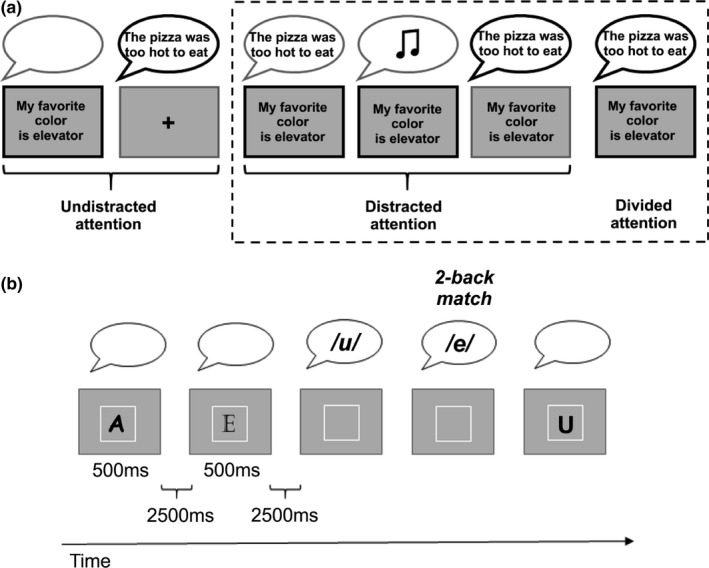
(a) A schematic illustration of the conditions of the attention task. The thicker black outlines denote which modality/modalities participants were instructed to attend to. The dashed box denotes those conditions that were included in the analyses of the current study (i.e., the distracted attention and divided attention conditions). The example sentences are the same for all conditions in the figure, but in the actual experiment, each sentence was presented only once to each participant. (b) A schematic illustration of the *n*‐back task showing five trials of the 2‐back condition including one vowel matching with a vowel delivered 2 trials back, and two modality switches

In the first part of the experiment, there were six experimental conditions, and one block of rest. The six different conditions of the attention task are depicted in Figure 1a. Of the six conditions, two were *undistracted attention* conditions, where sentences were presented only in the auditory (1) or visual (2) modality, and participants were instructed to attend to the presented sentence. Three conditions were *distracted attention* conditions demanding selective attention: the participants were instructed to attend to the sentences in just one modality and distractor stimuli were present in the other modality which the participants were instructed to ignore. Visual distractors were written sentences (3), and the participants were instructed to ignore them by holding a steady fixation on a cross presented in the middle of the screen. Auditory distractors were spoken sentences (4) or music (5). The final condition was the *divided attention* condition: the participants were presented with simultaneous spoken and written sentences (which differed in their content) and they were instructed to attend to both modalities (6). The sentences were presented for 2.5 s, after which the participants were instructed to respond whether the attended sentence was congruent or not (respectively), or during divided attention whether both attended sentences were congruent or whether one of the sentences had been incongruent. There were three functional runs, and each run included one rest block and one block of each task type, except the divided attention task was repeated twice. Each task block included 12 sentences (visual or auditory) or pairs of auditory and visual sentences.

After performing the attention task, anatomical images were acquired from the participants, and they then performed three levels of the *n*‐back task in separate blocks: 0‐, 1‐ and 2‐back. The design of the *n*‐back task is depicted in Figure 1b. In all three task levels, a vowel (a, e, u or y) was presented for 500 ms in either the visual or auditory modality, followed by a 2,500‐ms retention period. Depending on the *n*‐back task level, during the retention period participants were either asked to respond whether the presented vowel had been presented visually or auditorily (0‐back), or whether the vowel did or did not match the vowel presented *n* trials back irrespective of the modality of that vowel (1‐ and 2‐back). The modality of the presented vowel was switched randomly on every 3rd, 4th, 5^th^, or 7th vowel, so that participants were not able to anticipate a modality switch. There were two functional runs, and each run included one block of each *n*‐back task level. Each block included 32 vowels (visual or auditory). For more details on the procedures, please see our previous studies (Moisala et al., 2016, 2017).

### 2.5 Statistical analysis of behavioral data

Blocks where the percentage of correct responses was more than three standard deviations below average were removed from all further analyses, because during these blocks (with one block including trials of only one task condition), participants had most likely forgotten the task instructions and were performing the wrong task for the entire block. Performance accuracy for four Executive Task Types was analyzed: the total percentage of correct responses during (a) divided attention (DivA), (b) distracted attention (DistrA) and (c) 2‐back conditions (WM), as well as (4) the percentage of correct responses directly following a modality switch with trials from the 1‐ and 2‐back conditions combined (ModSwi). In the WM task type, only nonswitch trials (i.e., trials where the preceding and following vowels were presented in the same sensory modality) were included. Further, only the 2‐back task level was included in the WM task type because it taxed working memory more heavily than the 1‐back task. A repeated‐measures ANOVA was conducted for the percentage of correct responses with Executive Task Type as a within‐participant variable, Age Cohort and Gender as between‐participants factors, and GPA as a covariate of no‐interest.

Eta squared (*η* ^2^) was calculated for all conducted ANOVAs as a measure of effect size. For all conducted ANOVAs the Greenhouse‐Geisser *p*‐value was used (as indicated by the correction value *ε*), if the Mauchly's test of sphericity showed a significant result for a variable with more than two levels. However, original degrees of freedom will be reported with the *F*‐value even in these cases. A 95% confidence interval was used in all ANOVAs. When an ANOVA yielded a significant result, Bonferroni's post hoc tests were conducted. IBM SPSS Statistics 21 for Windows (IBM SPSS, Armonk, NY, USA) was used for statistical analyses.

### 2.6 Statistical analysis of fMRI data

Image preprocessing and statistical analysis was performed using Statistical Parametric Mapping (SPM12) analysis package (Wellcome Department of Cognitive Neurology, London, UK; Friston et al., 1994) as implemented in MATLAB. In order to allow for initial stabilization of the fMRI signal, the first four dummy volumes were excluded from analysis. In preprocessing, the slice timing was corrected, data were motion corrected, high‐pass filtered (cutoff at 1/128 Hz), and spatially smoothed with a 6‐mm Gaussian kernel. The EPI images were intra‐individually realigned to the middle image in each time series, and un‐warping was performed to correct for the interaction of susceptibility artifacts and head movements.

A mixed block/event‐related design was used for the tasks of the current study. This method allowed us to study both sustained fMRI effects and transient trial‐related activity (Petersen & Dubis, 2012). Trials belonging to the divided attention, distracted attention, and working memory conditions were clustered into blocks (but could also be modeled as separate events), but the switching condition included switch trials from both the 1‐ and 2‐back blocks. In the general linear model (GLM), all conditions were modeled as events instead of blocks. Fixed inter‐stimulus intervals were used, but as the TR was not synchronized with stimulus presentations, this effectively produced jitter in the sampling of the hemodynamic response function.

For the first‐level statistical analysis of the attention task, the GLM was set up including a regressor for each trial type. For the six different experimental conditions with speech or text sentences, a separate regressor was included for incongruent and congruent sentences. For each *n*‐back task level, separate regressors were used for trials preceding a modality switch, immediately following a modality switch, and for all other trials, and these regressors were further separated depending on whether the vowel was presented visually or auditorily. All data and regressors were estimated within a single GLM. Separate regressors for the responses of the participants and for instructions (2.5‐s periods between the blocks and a 6‐s period at the beginning of each run), as well as six movement parameters were added as nuisance regressors. The regressors were convoluted with the canonical hemodynamic response function. Similarly to the behavioral analyses, blocks where the percentage of correct responses was more than three standard deviations below average were removed from the analyses.

In the second‐level analysis, anatomical images were normalized to a canonical T1 template (MNI standard space) provided by SPM12 and then used as a template to normalize the functional images of each participant (tri‐linear interpolation, 3 mm × 3 mm × 3 mm using 16 nonlinear iterations). To study the effects of the different task conditions, statistical parametric maps (averaged across participants) were compared between contrasts for different task types and between contrasts for tasks and rest. A voxel‐wise height threshold was set at *t* = 2.7 and a cluster size threshold at *k* > 250. Anatomical regions corresponding to the activity foci were identified using the xjView toolbox for SPM (http://www.alivelearn.net/xjview).

### 2.7 Regional activity analyses

Four contrasts of interest were analyzed: activity during the (a) the divided attention condition compared with the distracted attention conditions (i.e., attention to speech with a text distractor and attention to text with a speech or music distractor), (b) the distracted attention conditions compared with the undistracted attention conditions (so that functional data from the undistracted attention to speech and undistracted attention to text conditions are combined), (c) the 1‐ and 2‐back conditions compared with the 0‐back condition (with only nonswitch trials included), and (d) trials in the 1‐ and 2‐back conditions immediately following a modality switch compared with trials preceding them. The contrasts were named Divided attention (DivA), Distracted attention (DistrA), Working memory (WM), and Modality switch (ModSwi), respectively. The four contrasts (*t* > 2.7, *k* > 250, cluster‐level Familywise error (FWE) corrected *p* < 0.01) were then overlaid on top of each other on an inflated cortical surface for each age cohort separately in order to visualize areas showing overlap between two or more contrasts. The average percentage of significantly active overlapping voxels between each contrast pair was then calculated for each age cohort separately. Statistical significance testings of the number of overlapping voxels were conducted based on contrasts in participant‐specific space. For this purpose, non‐normalized data were used in order to verify that any differences related to the normalization procedure between individuals would not affect the main findings. Although it is thought that MRI images of minors above the age of 6 and adults can be normalized to a common stereotactic space (Burgund et al., 2002), the impact of variability produced by individual brain maturation trajectories on the success of normalization procedures cannot be completely ruled out (Wilke, Schmithorst, & Holland, 2002). Even the youngest participants in the present sample had already reached middle adolescence and therefore marked childhood‐related distortions in normalization were unlikely. However, we still considered safer to conduct participant‐level analyses in non‐normalized space in order to produce more accurate results concerning age‐related differences. The advantage of using participant‐specific contrasts is also that any observed differences in contrast overlap between age cohorts could not be caused simply by greater variability in younger age cohorts. The participant‐specific contrasts were thresholded at a liberal level (*t* > 1.3, *k* > 100, uncorrected *p* < 0.10) in order to obtain sufficiently large activity clusters to produce overlapping regions between contrasts on a single participant level. Importantly, the analyses conducted on the uncorrected participant‐specific contrasts were only used as add‐on analyses to verify and confirm the main findings achieved by studying cluster‐corrected group‐level contrasts. The participant‐specific number of significantly activated voxels for each contrast, as well as the percentages of overlapping voxels between contrasts, was then subjected to a repeated‐measures ANOVA with Age Cohort and Gender as between‐participants factors, and GPA as a covariate of no interest.

### 2.8 Functional connectivity analyses

In order to determine regions‐of‐interest for functional connectivity analyses, regions showing overlap between all four executive task types across all three age groups were identified. To achieve this, first the four contrasts (DivA, DistrA, WM, ModSwi), which were thresholded for significance (*t* > 2.7, *k* > 250, cluster‐level FWE‐corrected *p* < 0.01), were overlaid on top of each other for each age cohort separately. A second‐level contrast was then derived for each age cohort, which included only those voxels showing overlap between all four contrasts. The resulting three‐second‐level contrasts (one per age group) were then overlaid on top of each other to reveal voxels where all four‐first‐level contrasts overlap in at least two of the age groups. Spheres with a radius of 7 mm were then set at the center of the clusters comprised of these voxels. This resulted in eight spherical regions‐of‐interest (ROIs) encompassing bilaterally the medial frontal gyrus (MFG), superior parietal lobule (SPL), the precuneus (Prc), and the medial supplementary motor area (SMA). The resulting spherical ROIs (i.e., *Executive ROIs*) were then used in ROI‐to‐ROI functional connectivity analyses. The functional connectivity was computed (separately for each participant) as a correlation between the average time course of signal intensity between each of the eight ROIs during task performance. All connectivity analyses were conducted using Conn software (Whitfield‐Gabrieli & Nieto‐Castanon, 2012).

The activation time course extracted for each participant for the functional connectivity analyses originated from the normalized and smoothed images. A standard denoising pipeline was applied to the data, where the six movement parameters, white matter, cerebrospinal fluid, as well as rest and task effects were added as confounding variables. Movement outliers were detected using the Artifact Detection Tools (ART) toolbox integrated into Conn, and the ART‐generated outliers were included as first‐level covariates and as confounding variables during denoising. Fisher's *r* to *z* transformation was applied to the resulting correlation coefficients for each participant. The participant‐level beta values for the correlation between the average time course of signal intensity between each of the eight ROIs during the four executive task types were then extracted.

Next, differences in functional connectivity between the executive task types and between age cohorts were examined. The connectivity analysis of eight ROIs resulted in 28 ROI‐to‐ROI connectivity values. We calculated the correlations between these ROI‐to‐ROI connectivity values across the four executive task types for each participant. The resulting correlation matrices were averaged across all participants in order to examine general task‐related functional connectivity, and across participants within each age cohort in order to examine whether age affected the functional connectivity between the four executive task types. Correlations (of correlation matrices) between age cohorts were calculated, and the resulting correlation matrices were visualized using multidimensional scaling (RSA toolbox; Nili et al., 2014). Euclidian distances were calculated for all ROI‐to‐ROI pair connections (data averaged across participants within an age cohort) and compared between the three age cohorts by conducting a univariate ANOVA with Age Cohort and Connectivity Type as the fixed factors. A similar univariate ANOVA was also conducted for the Euclidian distances with Age Cohort and Connectivity Distance (i.e., long vs. short range connections) as the fixed factors. Short range connections were defined as SPL‐to‐Prc or MFG‐to‐SMA connections within one hemisphere, and all other connections were defined as long range connections.

### 2.9 Measuring and controlling for head motion

Head motion can have a significant impact on developmental fMRI studies, since minors tend to move more than adults. Excessive head motion is known to produce artifacts (Friston, Williams, Howard, Frackowiak, & Turner, 1996) and can therefore affect fMRI results, especially in terms of functional connectivity in developmental studies (Van Dijk, Sabuncu, & Buckner, 2012).

Several measures were taken to minimize effects of head motion on fMRI data in the current study. Firstly, participants with persistent excessive head motion (greater than ±3 mm of head motion in over 25% of scans) were discarded from the analyses (*n* = 2). For the final 167 participants of this study, a framewise data quality index DVARS was calculated for each participant (separately for the attention and working memory tasks) and compared between age cohorts using a multivariate ANOVA with Age Cohort and Gender as between‐subjects variables. DVARS is a measure of the rate of change of blood‐oxygenetion level‐dependent (BOLD) signal across the entire brain at each frame of data (i.e., how much the intensity of a brain image changes in comparison to the previous timepoint), and it is used to detect and control for motion artifacts in fMRI data, especially in relation to functional connectivity analyses (Power, Barnes, Snyder, Schlaggar, & Petersen, 2012; Power et al., 2014). Importantly, when the DVARS values for the attention and working memory tasks were compared between age cohorts, no main effect of Age Cohort was observed for either the attention or working memory task runs (*p* = 0.32 and *p* = 0.17, respectively). When examining regional fMRI activity, the source of signal caused by movement artifacts was modeled by including the six motion parameters as covariates of no interest in the GLM (Friston et al., 1996; Johnstone et al., 2006). For the functional connectivity analyses, the six movement parameters along with white matter and cerebrospinal fluid were also added as confounding variables. In addition, movement outliers were detected using the Artifact Detection Tools (ART) toolbox integrated into Conn, and the ART‐generated outliers were included as first‐level covariates and as confounding variables during denoising. Together, these data quality control measures minimized the impact of head motion on the present fMRI data.

## 3 RESULTS

The ages and grade point averages (GPAs) per age cohort and gender are displayed in Table 1.

### 3.1 Behavioral results

For the attention task, in 33 of 3,612 blocks the percentage of correct responses was three standard deviations lower than the mean (below 37.2%), and these blocks were excluded from further analyses. For the *n*‐back task, in 32 of 1,020 blocks the percentage of correct responses was three standard deviations lower than the mean (below 57.2%), and these blocks were excluded. The number of discarded blocks in the attention task did not demonstrate a main effect of Age Cohort (*p* = 0.66). For the *n*‐back task, a main effect of Age Cohort was observed (*F*(2,166) = 4.66, *p* = 0.01, *η* ^2^ = 0.05), and post hoc tests confirmed that there were more discarded blocks in the middle than in the late adolescent age cohort (5.85% vs. 1.47% of discarded blocks, respectively; *p* = 0.01).

Correlations in performance accuracy between the four executive task types are listed in Table 2.

**Table 2:** Correlations in performance accuracy between the four executive task types

	13–14 years	16–17 years	20–24 years	Overall
*r*_DivA DistrA_	0.66**	0.64**	0.47**	0.61**
r_DivA WM_	0.08	0.10	0.20	0.12
*r*_DivA ModSwi_	0.44**	0.08	0.13	0.25**
*r*_DistrA WM_	0.08	0.28*	−0.18	0.09
*r*_DistrA ModSwi_	0.35*	0.10	−0.16	0.18*
*r*_ModSwi WM_	0.53**	0.56**	0.44**	0.52**

*Notes*

Partial correlations between task performances for each of the four executive task types are displayed separately for the three age cohorts, along with an overall correlation between task types. Correlations are controlled for Gender in all analyses, and also for Age Cohort when calculating the overall correlation.

DivA: Divided attention; DistrA: Distracted attention; ModSwi: Modality switch; WM: Working memory.

Significant correlations are marked with asterisks (*p < 0.05, **p < 0.005).

**Figure 2:**
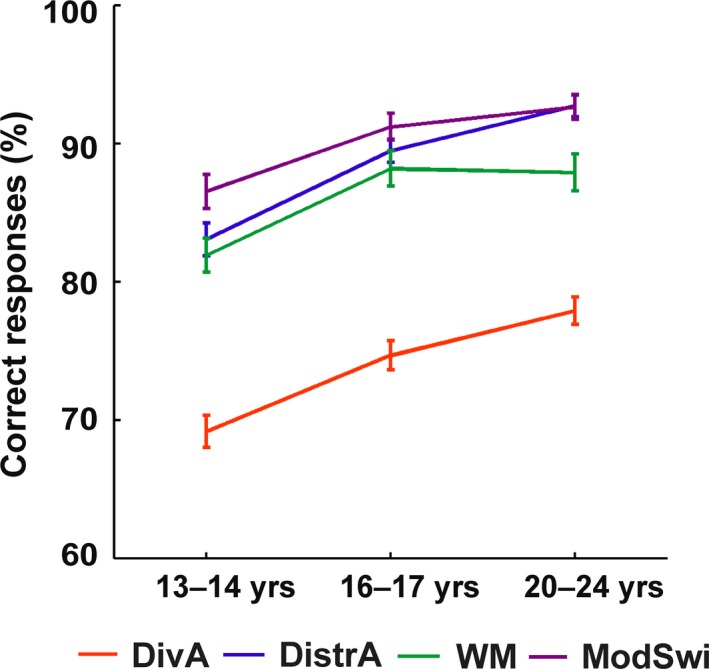
The percentage of correct responses separately for each executive task type and age cohort. Error bars show standard errors of the mean. DivA: Divided attention; DistrA: Distracted attention; ModSwi: Modality switch; WM: Working memory

Figure 2 depicts the percentage of correct responses for each executive task type for each age cohort separately. A main effect of Executive Task Type was observed (*F*(3,480) = 9.26, *p* < 0.001, *η* ^2^ = 0.05, *ε* = 0.76). Performance accuracy decreased from ModSwi (*M* = 90.08, *SD* = 8.24) to DistrA (*M* = 88.33, *SD* = 8.29) and further to WM (*M* = 86.23, *SD* = 9.72) and finally to DivA (*M* = 73.82, *SD* = 8.78). All other pairwise comparisons between executive task types were significant (*p* < 0.001), apart from the difference between DistrA and WM (*p* = 0.05) as well as DistrA and ModSwi (*p* = 0.14). Of the between‐participants variables, Age Cohort had a main effect on performance (*F*(2,160) = 24.81, *p* < 0.001, *η* ^2^ = 0.23). The middle adolescent cohort performed worse than the two older age cohorts (*p* < 0.001 for both), but no difference was observed between the late adolescent and young adult cohorts (*p* = 0.08). The interaction between Age Cohort and Executive Task Type was not significant (*p* = 0.10). Gender demonstrated a main effect (*F*(1,160) = 4.17, *p* = 0.04, *η* ^2^ = 0.02), with females performing better that males (*M* = 85.64, *SD* = 8.12 and *M* = 83.83, *SD* = 7.83, respectively). No interaction between Age Cohort and Gender was observed (*p* = 0.20). GPA also demonstrated no main effect on performance (*p* = 0.11).

### 3.2 fMRI results

**Figure 3:**
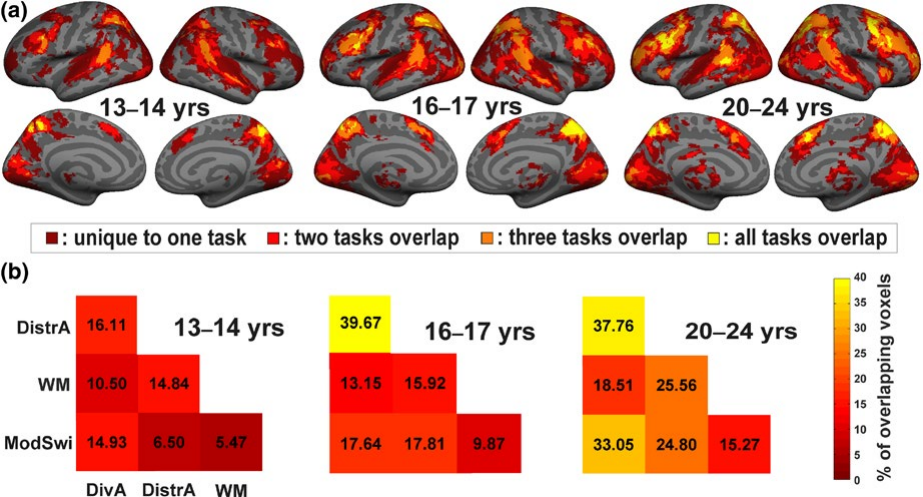
(a) Voxels showing significant activity enhancements (*t* > 2.7, *k* > 250, cluster‐level FWE‐corrected *p* < 0.01) in response to one or more of the executive tasks, with the color of the voxel denoting how many of the executive tasks significantly activate that voxel. Dark red regions: activity enhancements for only one executive task, bright red regions: two executive tasks overlap, orange: three executive tasks overlap, yellow: all four executive tasks overlap. The maps are plotted for each age cohort separately. (b) The percentage of overlapping, significantly activated voxels between each of the executive task types is visualized in a matrix for each age cohort separately. DivA: Divided attention; DistrA: Distracted attention; ModSwi: Modality switch; WM: Working memory

fMRI analyses were first used to determine how cortical networks recruited by the different executive task types differ between the three age cohorts. Figure 3 shows the overlap between cortical regions activated by the four executive task types for each age cohort separately. In all three age cohorts, the cortical network activated by at least one of the executive task types activated a large cortical network comprising of the auditory and visual cortices, dorsolateral prefrontal cortex, medial superior frontal gyrus, superior parietal lobule, temporo‐parietal junction and precuneus (Figure 3a). However, when the degree of overlap in the regions activated by the executive task types are studied, differences emerged between the age cohorts. In the two younger age cohorts, areas significantly activated by all four executive task types (yellow areas in Figure 3a) included the dorsolateral prefrontal cortex (Brodmann area, BA 6/8/9) mostly only in the left hemisphere, the superior parietal lobe (BA 7) again more prominently in the left hemisphere, and the precuneus (BA 7) bilaterally. For the oldest age cohort, these regions extended to include much more of the cortex: the dorsolateral prefrontal cortex (BA 6/8/9) extending bilaterally to now include portions of the superior, middle and inferior frontal gyri, the superior parietal lobe (BA 7), the posterior portion of the superior temporal gyrus (BA 22/41/42) in the left hemisphere, the temporo‐parietal junction (BA 39), the precuneus (BA 7), and medial superior frontal gyrus (BA 6) in the right hemisphere. The percentage of overlapping voxels for each executive task pair ranged from 5.5% to 16.1% in the middle adolescent cohort, from 9.9% to 39.7% in the late adolescent cohort, and from 15.3% to 37.8% in the young adult cohort (Figure 3b).

In order to confirm that differences in the normalization procedure for fMRI data did not affect our results, the previous ROI analyses were repeated for contrasts defined in participant‐specific space. Firstly, these analyses revealed that the overall number of significantly activated voxels for each of the four executive task types was not significantly affected by Age Cohort, although a nonsignificant trend was observed (*p* = 0.054) so that according to the post hoc test the amount of significantly active voxels was lower overall for the middle adolescent cohort than for the young adult cohort (*p* = 0.048). No interaction between Age Cohort and Executive Task Type was observed, either (*p* = 0.47). However, when the percentage of significantly active overlapping voxels was studied, a main effect of Age Cohort (*F*(2,160) = 5.94, *p* = 0.003, *η* ^2^ = 0.07, *ε* = 0.82) was observed. Pairwise post hoc comparisons revealed that the percentage of overlapping voxels in participant‐specific space was significantly lower for the middle adolescent cohort than the young adult cohort (*p* = 0.002). There was no significant difference between the middle adolescent cohort and the late adolescent cohort (*p* = 0.18). Given that there were more discarded blocks in the middle adolescent than late adolescent cohort due to poorer behavioral performance, the analyses of percentages of overlapping voxels in participant‐specific space were repeated so that the worst performing participants were discarded from the analyses. This was performed to ensure that including different amounts of blocks for different participants would not affect the participant‐level (i.e., nonaveraged) results. Thus, participants with less than two blocks of any task type were discarded, corresponding to 14 participants from the middle adolescent cohort, 4 from the late adolescent cohort, and 6 from the young adult cohort. Analyses with this pruned dataset were found to mirror the results conducted on the full dataset, so that the main effect of Age Cohort was nearly significant (*F*(2,137) = 3.01, *p* = 0.05, *η* ^2^ = 0.04, *ε* = 0.73), and post hoc comparisons revealed that the percentage of overlapping voxels was significantly lower for the middle adolescent cohort than the young adult cohort (*p* = 0.03).

**Figure 4:**
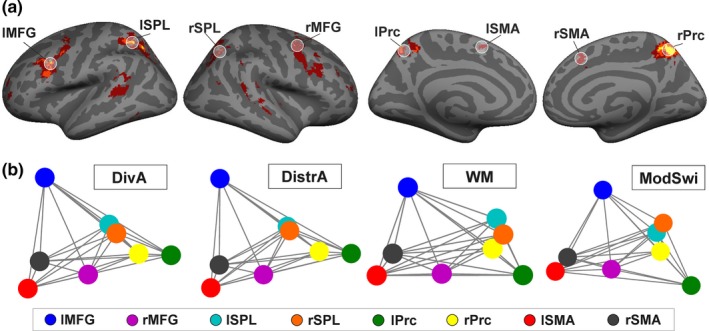
(a) Voxels showing significant activity enhancements (*t* > 2.7, *k* > 250, cluster‐level FWE‐corrected *p* < 0.01) in response to all four executive tasks in only one age cohort (red), in two age cohorts (orange) or in all three age cohorts (yellow). Executive regions‐of‐interest (ROIs) with a radius of 7 mm are denoted with white transparent circles. These ROIs best captured the clusters comprised of voxels activated by all four executive task types in at least two of the age cohorts. (MFG: middle frontal gyrus; Prc: precuneus; prefix r: right hemisphere; prefix l: left hemisphere; SMA: supplementary motor area; SPL: superior parietal lobe). (b) The four correlation matrices for ROI‐to‐ROI functional connectivity between each of the Executive ROIs (one per each executive task type) correlated significantly with one another (*p* < 0.001 in all cases). The four ROI‐to‐ROI functional connectivity matrices are visualized here using multidimensional scaling, so that the distances between two nodes reflect the degree of observed functional connectivity between the ROIs across all participants (i.e., shorter distances reflect stronger functional connectivity). DivA: Divided attention; DistrA: Distracted attention; ModSwi: Modality switch; WM: Working memory

In addition to analyses of regional cortical activity, functional connectivity between key task‐positive cortical hubs was also examined. Figure 4 depicts the observed general task‐related functional connectivity results. Functional connectivity was examined specifically between eight frontoparietal ROIs (i.e., *Executive ROIs*) that were activated by all four executive task types in at least two of the age cohorts (Figure 4a). These regions comprised of the right MFG (ROI center in MNI coordinates *x*,* y*, and *z*: 36, 2, 50), left MFG (−37, 13, 34), right medial SMA (rSMA; 9, 20, 47), left medial SMA (−7, 9, 53), right Prc (8, −62, 52), left Prc (−13, −56, 50), right SPL (33, −62, 45), and left SPL (−26, −59, 50). Functional connectivity between the *Executive ROIs* during each of the four executive task types is visualized using multidimensional scaling, so that the distances between two nodes reflect the degree of observed functional connectivity between the ROIs across all participants (Figure 4b). The four correlation matrices correlated significantly with one another (*p* < 0.001 in all cases), demonstrating no significant difference in the pattern of functional connectivity between the four executive task types when data were averaged across participants.

**Figure 5:**
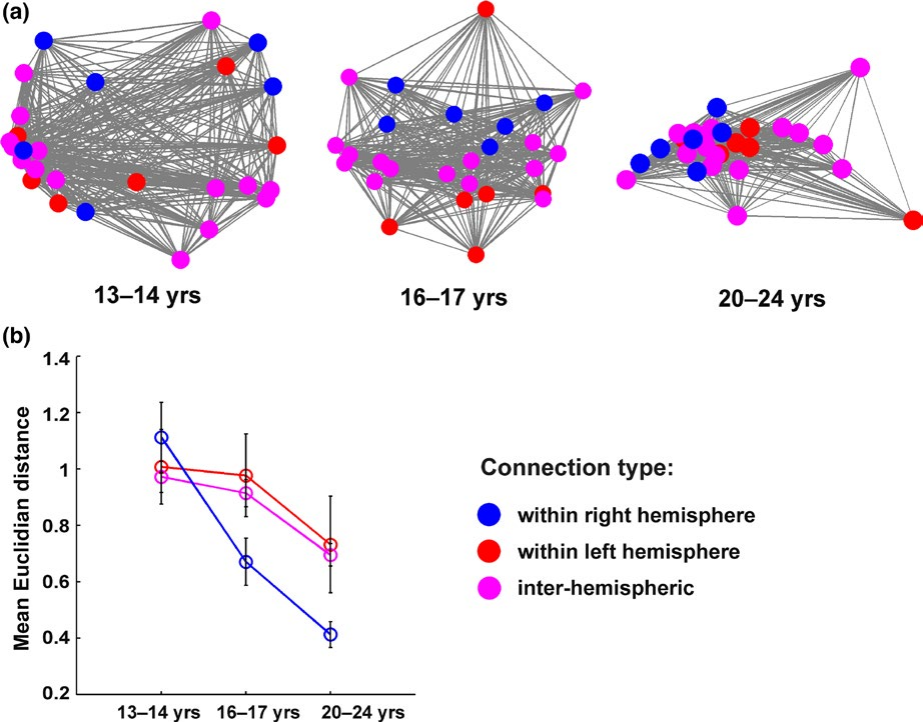
(a) Correlation matrices for the connectivity across all ROI‐to‐ROI pairs (28 in total) between the four executive task types are visualized for each age cohort using multidimensional scaling. Each node depicts one ROI‐to‐ROI connection, and the distance between two nodes reflects the magnitude of correlation between the functional connectivity beta values of two ROI pairs across all four task types (i.e., shorter distances reflect a stronger correlation). All three correlation matrices differed from each other significantly (all *p*s < 0.05). (b) The average Euclidian distances between ROI‐to‐ROI pair connections are depicted for each age cohort. Error bars show standard errors of the mean. Colors denote the type of connection: red denotes connections within the right hemisphere, blue denotes connections within the left hemisphere, and magenta denotes inter‐hemispheric connections

Next, we examined whether the three age groups differ in how similar the functional connectivity between the *Executive ROIs* was between the four different executive task types. This question was approached by first calculating correlation matrices by cross‐correlating ROI‐to‐ROI activity between the four task types, and then determining whether the correlations differed significantly between the three age groups. The three resulting correlation matrices are visualized using multidimensional scaling in Figure 5a. Each node depicts one ROI‐to‐ROI connection, so that red nodes denote connections within the right hemisphere, blue nodes denote connections within the left hemisphere, and magenta nodes denote inter‐hemispheric connections. The distance between two nodes reflects the magnitude of correlation between the functional connectivity beta values of two ROI pairs across all four task types (i.e., shorter distances reflect a stronger correlation). In other words, the more spread out the nodes are, the more dissimilar the general pattern of functional connectivity between the four different executive tasks within the age group. The results show that the similarity of connectivity across tasks increases with age. All three correlation matrices differed significantly from each other (*p* < 0.05 for all comparisons). Figure 5b depicts the mean Euclidian distances between the nodes for each age cohort separately. The same color scheme is used to denote connection types in Figure 5b as in Figure 5a. The ANOVA conducted for the mean Euclidian distance values demonstrated a Bonferroni‐corrected main effect of Age Cohort (*F*(2,441) = 10.36, *p* < 0.001, *η* ^2^ = 0.04). Pairwise comparisons revealed that the young adult cohort had significantly smaller Euclidian distance values than the middle adolescent (*p* < 0.001) or late adolescent cohort (*p* = 0.03) across all connection types. The difference between the middle and late adolescent cohorts was not significant (*p* = 0.17). Connectivity Type (i.e., connectivity within the left vs. right hemisphere vs. inter‐hemispheric connectivity) did not demonstrate a main effect (*p* = 0.23), nor did it interact significantly with Age Cohort (*p* = 0.24). Moreover, Connectivity Distance (long vs. short range connections) did not have a significant main effect (*p* = 0.13), and there was no significant interaction of Connectivity Distance and Age Cohort (*p* = 0.45).

## 4 DISCUSSION

In the current study, age‐related improvements in executive functioning were observed between the youngest and the two older age cohorts. That is, the late adolescent and young adult participants made significantly less errors than the youngest participants irrespective of the specific nature of the executive task type. This was to be expected, as cognitive development is known to continue throughout adolescence, especially with regard to executive functioning (for reviews, see Paus, 2005; Zelazo & Müller, 2002). The present results add to this body of evidence by demonstrating that significant improvements in cognitive performance occur between the ages of 13 and 24 years in several measures of executive functioning. A further behavioral finding was that task performances between the four executive task types correlated significantly. Similarly, age‐related improvements in task performance did not differ between the task types. That is, fewer errors were made in the late adolescent and young adult age groups irrespective of whether the task required divided attention, selective attention and inhibition of distractors, working memory, or modality switching. Some previous studies have found that different aspects of executive function mature at different rates during adolescence (Anderson, Anderson, Northam, Jacobs, & Catroppa, 2001), but our results imply that more general aspects of executive functioning mature during this time. In sum, our behavioral findings suggest that (a) different measures of executive functioning rely, at least to some extent, on a domain‐general capacity which is already evident during middle adolescence and that (b) this capacity matures from middle adolescence to late adolescence and early adulthood, producing uniform improvements across different executive task types. It should be noted, however, that the experimental tasks used in the current study were designed to be performed during an fMRI measurement. More finely tuned paradigms designed specifically for behavioral studies (e.g., with more trial repetitions) might have been more optimal to tease apart different cognitive processes and could have produced smaller correlations between the different task types. The four executive subfunctions targeted in the present study were measured with only two task paradigms. This may have also contributed to the correlations in performance between the different task conditions, and affected the conclusion that more general aspects of cognitive functioning mature during adolescence.

It should be also noted that the tasks used in the current study were not typical for studies examining different sub‐processes of executive function. When studying divided attention, for example, more simplistic stimuli are often used, such as tones and geometric shapes (e.g., Johnson & Zatorre, 2006). The divided attention task used in the current study was designed to be more ecologically valid than the more standard task paradigms, which increased the complexity of the task and introduced semantic processing to the task requirements. The task has, however, been validated in our previous study with young adult participants (Moisala et al., 2015), where it was shown to produce performance outcomes and brain activity patterns similar to those in previous studies on divided attention.

Moreover, the distracted attention task used in the current study was also not a classical measure of inhibition. Cognitive inhibition is most often measured with tasks such as Stroop and go/no‐go, which involve inhibiting a prepotent response. Again, these more established measures often involve simple stimuli, whereas our aim was to induce the need to inhibit processing of a to‐be‐ignored stimulus in a more ecologically valid task setting. Furthermore, the inhibitory control component of executive functions is not thought to involve only response inhibition, but also interference control (i.e., selective attention and cognitive inhibition) (Diamond, 2013; Lehto et al., 2003; Miyake et al., 2000). Our distracted attention task can therefore be argued to tap into inhibitory control processes even though it did not require response suppression in the classical sense, as it involved selective attention to a stimulus while suppressing a to‐be‐ignored stimulus.

Finally, with regard to the modality switching condition used in the current study, this task did not involve the classical change in stimulus‐response rules required by most other task switching paradigms. Switching (or shifting) is defined as shifting back and forth between multiple tasks, operations, or mental sets. In our task, participants had to switch between matching visually presented letters to matching auditorily presented letters. The stimulus‐response rule in itself (i.e., respond whether or not the letter matched) did therefore not change, but the stimulus category/properties did. Previous studies have used similar single‐task designs requiring cross‐modal attention shifts, and they have demonstrated similar performance decrements as in traditional switching paradigms with two different tasks within one modality (Lukas, Philipp, & Koch, 2010; Spence & Driver, 1997). In our previous study using this same task, we showed that the modality switch caused performance decrements reflected by both prolonged response times and increased number of response errors (Moisala et al., 2017). It has nevertheless been argued that the control processes mediating task switching and modality switching seem to be separable to some extent (Hunt & Kingstone, 2004; Murray, De Santis, Thut, & Wylie, 2009), and that different types of switching have been found to activate largely overlapping but also partially separable cortical networks (Kim, Cilles, Johnson, & Gold, 2012). It is therefore possible that using two different tasks in the same modality, instead of using an intermodal single‐task design, could have produced differing results in the current study.

Although executive functions are known to mature during adolescent development, and to rely at least partly on a more general aspect of cognitive functioning, relatively little is known about how these two phenomena are reflected in patterns of brain activity. In the current study, we examined both cortical activity and functional connectivity. Significant age‐related differences in both indices were observed during executive task performance. Firstly, the overlap between cortical regions recruited by the different task types was observed to increase with age when comparing the middle adolescent cohort to the young adult cohort. In the 13‐ to 14‐year‐olds, the highest group average of the percentage of overlapping voxels observed between two task types was around 19%, whereas for the 16‐ to 17‐year‐olds and 20‐ to 24‐year‐olds, it was around 38%. Among the middle and late adolescent participants, the brain areas activated by all four executive task types included only the dorsolateral prefrontal cortex, superior parietal lobe, and precuneus. For the young adults, these regions extended to include much more of the cortex in the dorsolateral prefrontal cortex, superior temporal gyrus extending to the temporo‐parietal junction, and the medial superior frontal gyrus. A plethora of previous studies on adults have confirmed the importance of these very same cortical regions for executive functioning. It is commonly held that a “frontoparietal control network” is crucial to all executive processes (Badre & D'Esposito, 2007; Fedorenko et al., 2013; Vincent et al., 2008). In addition to this shared brain circuitry, brain imaging studies have repeatedly observed a differential functional organization or unique response patterns within shared brain regions between different executive control components (Marklund et al., 2007; Miyake et al., 2000; Stiers, Mennes, & Sunaert, 2010; Wager & Smith, 2003). It is therefore likely that the neural networks recruited by executive functions demonstrate both unity and diversity (Friedman & Miyake, 2017). The current study adds a pivotal contribution to previous research by demonstrating that the homogeneity of neural recruitment between different executive processes may, in fact, increase with age.

A similar pattern of developmental unification emerged when functional connectivity between key frontoparietal hub regions was examined. More specifically, connectivity patterns during the performance of different executive tasks were less distinguishable in the young adult cohort than in the two adolescent cohorts. This was true both for intrahemispheric and interhemispheric connections. When the data were averaged across all age cohorts, in line with our initial hypothesis, the pattern of functional connectivity was not found to differ significantly between the four executive task types. That is to say, the similarity of frontoparietal functional connectivity was not affected by the specific nature of the task themselves, but rather by the age of the participant. A vast amount of previous research has shown that functional connectivity changes with age. These studies have shown, for example, that long‐range connectivity strengthens and short‐range connectivity weakens with age (Dosenbach et al., 2010; Fair et al., 2007; Supekar, Musen, & Menon, 2009), and that connectivity between regions belonging to the “default‐mode network” become more cohesive (Fair et al., 2008; Supekar et al., 2010) and integration between networks recruited by cognitive control increases with age (Stevens et al., 2007; Supekar & Menon, 2012; Washington & VanMeter, 2015). Contrary to these previous studies, our aim was not to study changes in the strength of functional connectivity during executive processing per se. Instead, we examined the correlations between patterns of task‐related functional connectivity. This way we were able to reveal a striking age‐related difference in the similarity of functional connectivity between different executive task types. Future studies utilizing more exploratory seed‐to‐voxel analyses may produce an even more comprehensive understanding of age‐related changes in functional connectivity.

**Figure 6:**
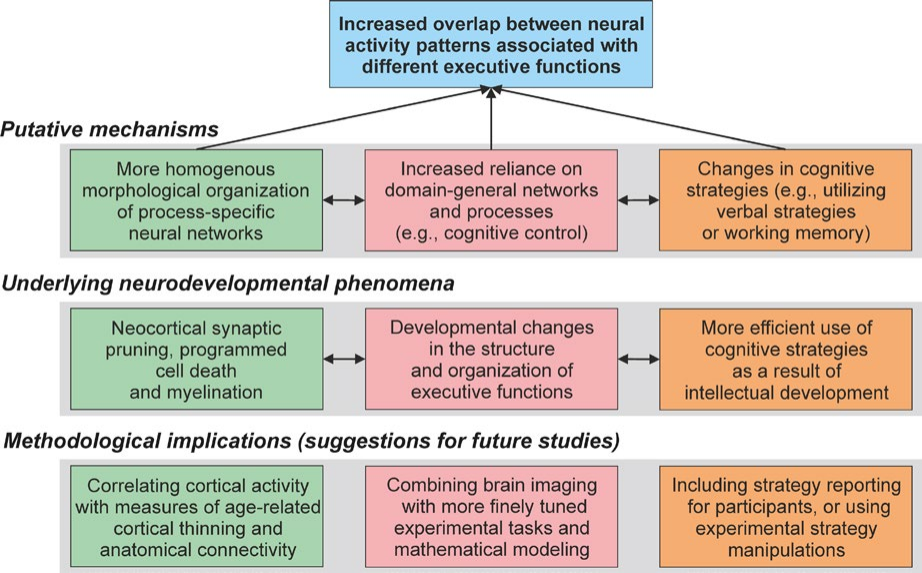
A summary of the theoretical and methodological implications of the findings of the current study

A summary of the theoretical and methodological implications of the current study is presented in Figure 6. Several tentative hypotheses regarding the neurocognitive mechanisms underlying our findings can be formed. First, cortical activity and functional connectivity may become more consistent with age due to a more homogenous morphological organization between process‐specific neural networks. Although previous studies have shown that different executive tasks elicit activity in partly distinct neural networks (Miyake et al., 2000; Wager & Smith, 2003), our results are the first ones suggesting that these process‐specific networks seem to become morphologically less separable with age. Such a restructuring of neural networks is most likely a result of synaptic pruning, programmed cell death and myelination, which are known to occur during neural development (Blakemore, 2012; Huttenlocher & Dabholkar, 1997; Liston et al., 2006). Particularly during adolescence, activity of long‐term depression mechanisms is increased, leading to heightened synaptic elimination (Selemon, 2013). Although previous studies provide insight into the molecular mechanisms of *how* synaptic pruning occurs during development, our findings reveal *which specific networks* it is likely to target. Our results suggest that this synaptic pruning occurs mostly in divergent, process‐specific neural networks, whereas connections in circuits recruited by a wide variety of cognitive processes are less affected. Since connections in domain‐general networks are recruited more often than connections related to specialized aspects of cognitive processes, they are more likely to succeed in a Hebbian activity‐dependent competition between neuronal connections (Shatz, 1990), This, in turn, may lead to a gradual morphological re‐organization of cortical networks related to executive processes. Selective pruning in process‐specific networks may also explain why some studies have found that children exhibit more diffuse neural activity than adults (Casey et al., 2000; Durston et al., 2006; Geier et al., 2009). Our findings regarding functional connectivity may also be explained by neural maturation. As diffusion becomes more restricted with age in frontal pathways (Asato, Terwilliger, Woo, & Luna, 2010; Liston et al., 2006), functional connectivity patterns may become more consistent across different cognitive tasks, as suggested by the current study. In order to further explore the relationship between age‐related morphological reorganization and neural recruitment, future studies should directly compare measures of cortical activity and connectivity with indices of anatomical neural maturation (e.g., cortical thinning and axonal myelination).

An alternative explanation for our results is that cognitive development may result in increased reliance on a more domain‐general processes involved in executive processing. In other words, different executive functions may depend on more task specific processes (e.g., shifting, inhibition) and therefore recruit more specialized regions during adolescence, whereas adults recruit areas related to more generalized processes (i.e., cognitive control). This means that the present fMRI findings may not reflect development changes in brain processes themselves, but rather age‐related differences in psychological processes and cognitive architecture. Although some studies have suggested that the architecture of executive functions changes during cognitive development, there does not seem to be much support for the notion that different executive functions come to rely more heavily on a common cognitive function with age. On the contrary, the results of Brydges et al. (2014) suggest that during childhood development, executive functions seem to shift from a unitary control process toward a more differentiated function. They studied the performance of 135 children (mean age ca. 8 years) in a wide range of executive tasks and repeated the tests 2 years later. They found that a one‐factor model of executive functions changed to a two‐factor model between the two measurement points. Other researchers have argued that different sub‐processes of executive functioning remain separate throughout development, but that their expression and relationships vary with age (Demetriou & Spanoudis, 2015). Further studies would benefit from combining brain imaging with finely tuned experimental paradigms aimed to behaviorally tease apart different executive processes, and structural equation modeling to determine the factor structure of performance data. This way, analysis of brain activity patterns could be used to complement behavioral findings concerning the cognitive architecture of executive processes. It should be noted, however, that an increase in the degree of overlap between brain regions recruited by different executive processes does not necessarily imply that they become more dependent upon a common process. This is because functionally separate networks may comprise of increasingly overlapping regions of the cortex, since separate networks can involve the same single voxel but may not be separable with fMRI due to limitations in the spatial resolution of this method.

Finally, developmental changes in cognitive strategy may also contribute to the current findings and to developmental fMRI studies in general (Crone & Steinbeis, 2017; Luna, Padmanabhan, & O'Hearn, 2010). For example, the young adult participants may have consistently used cognitive strategies that rely more on working memory, thus producing more consistent patterns of neural recruitment across tasks. For example, the superior performance of older participants in the modality switching task may have been due to more efficient maintenance of the presented stimulus in working memory following the modality change. Adults may also rely more on verbal strategies (such as verbal rehearsal) than younger individuals to accomplish cognitive tasks (Van Leijenhorst, Crone, & Van der Molen, 2007), again affecting neural recruitment during task performance. Using verbal strategies may have been especially likely in the present study, given that the present stimuli consisted of letters and words. Future studies would benefit from thoroughly investigating the task strategies used by the participants and comparing brain activity associated with different strategies within and between age groups. It should also be noted that the current participants represented a selective sample of relatively high‐functioning individuals (e.g., university students and pupils with relatively high GPAs), who perform well academically and thus are likely to use cognitive strategies effectively. The present study nevertheless provides important and novel insight into adolescent brain development by suggesting that increasing homogeneity in cortical recruitment across different executive tasks may be responsible for age‐related improvements in executive skills. Studies utilizing longitudinal data are needed to more precisely disentangle complex interactions between age‐related changes in cognitive functioning and neural recruitment.

## 5 CONCLUSION

Brain imaging studies focusing on the development of neural and cognitive architectures during adolescence are scarce, even though marked improvements in executive functioning are known to occur during this time. The current study offers novel insight into the neural basis of age‐related improvements in executive functioning by showing that the similarity of neural responses elicited by different executive processes increases from adolescence to early adulthood. This result provides a possible neural basis for age‐related behavioral improvements in executive functioning during adolescence, significantly furthering our understanding of normative brain maturation during this important developmental phase.
